# Toxoplasmic Lymphadenitis Presenting as a Tiny Neck Tumor

**DOI:** 10.3390/healthcare9050487

**Published:** 2021-04-21

**Authors:** Shih-Lung Chen, Jim-Ray Chen, Shih-Wei Yang

**Affiliations:** 1 Department of Otolaryngology & Head and Neck Surgery, Chang Gung Memorial Hospital, Linkou 333, Taiwan; rlong289@gmail.com; 2 School of Medicine, Chang Gung University, Taoyuan 333, Taiwan; 3 Department of Pathology, Keelung Chang Gung Memorial Hospital, Keelung 204, Taiwan; jimrchen@cgmh.org.tw; 4 Department of Otolaryngology & Head and Neck Surgery, Chang Gung Memorial Hospital, Keelung 204, Taiwan

**Keywords:** toxoplasmic lymphadenitis, ultrasound, fine needle aspiration cytology, head and neck

## Abstract

(1) Background: Toxoplasmic lymphadenitis (TL), caused by the protozoan *Toxoplasma gondii*, is a worldwide zoonosis. We report a case of TL in the head and neck region diagnosed using ultrasound (US)-guided fine needle aspiration cytology (FNAC), serological tests, and pathological findings. (2) Case Presentation: A 51-year-old female with a chief complaint of a left posterior neck mass that had been growing for approximately 2 weeks. TL was confirmed by histopathological examinations and serological tests. US-guided FNAC and en bloc resection of the lymph node were performed. The diagnosis was confirmed as TL in the neck. (3) Conclusions: We suggest that US-guided FNAC should be considered as the first-line test for assessing a tiny mass before a definitive treatment is chosen.

## 1. Introduction

The prevalence of toxoplasmosis is increasing globally. In Taiwan, the overall seroprevalence of *Toxoplasmic gondii* (*T. gondii*) infection is 9.3% in the major regions [1]. Toxoplasmic lymphadenitis (TL) caused by *T. gondii* primarily involves the neck and occipital areas. Other frequent sites include the axillary, submental, inguinal, and supraclavicular lymph nodes, and ocular and cerebral areas [2]. Here, we describe a patient who presented with a painless, enlarged mass in the left posterior neck. We used ultrasound (US)-guided fine needle aspiration cytology (FNAC) and surgical intervention to approach the mass lesion, and confirm the diagnosis of TL.

## 2. Case Presentation

**Figure 1:**
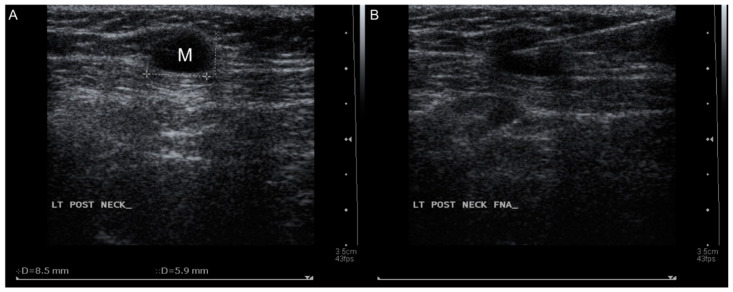
(**A**) Targeted ultrasound revealed an ovoid hypovascular hypoechoic mass with a short axis of about 0.6 cm and preserved. (**B**) Fine needle aspiration cytology was performed using a 21-gauge needle.

**Figure 2:**
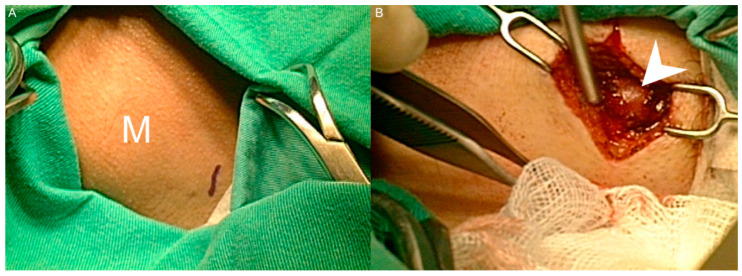
(**A**) A well-defined and soft mass was noted in the left posterior neck. No erythematous change or local heat over the skin was observed. (M: mass). (**B**) The incision was made above the mass. The left neck mass (arrowhead) was explored and en bloc resection was conducted.

**Figure 3:**
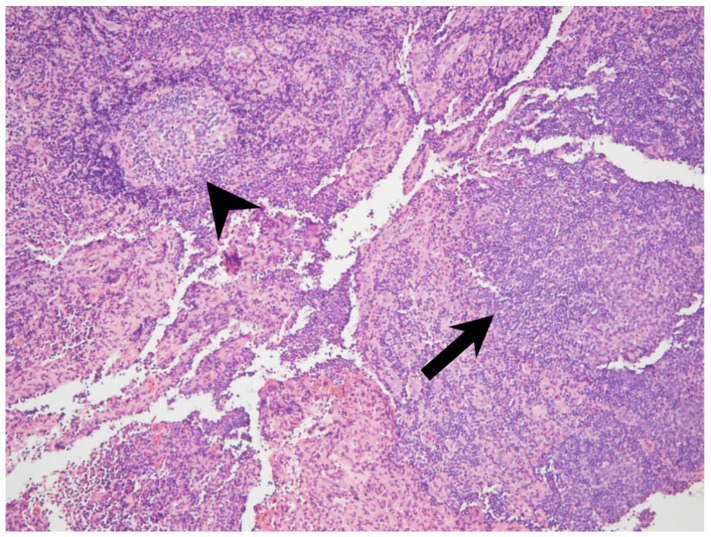
Microscopic examination revealed reactive follicular hyperplasia (arrowhead) and the presence of small clusters of epithelioid histiocytes (arrow) in the affected lymph node (original magnification ×40).

A 51-year-old female came to our department with a chief complaint of a left posterior mass that had been growing for approximately 2 weeks. The patient had no fever, night sweats, weight loss, traumatic episode, surgical history, or painful sensations, and no specific medical disease or systemic disorder. She had owned a dog for 3 years. A physical examination revealed a well-defined mass in the left posterior triangle of her neck. Laboratory findings showed a normal C-reactive protein level (4.80 mg/L; normal: <5 mg/L) without leukocytosis (9500/µL; normal: 3900–10,600/µL). To differentiate this mass lesion, we arranged US-guided FNAC. Target US revealed an ovoid hypovascular hypoechoic lymph node with a short axis of about 0.6 cm and preserved fatty hilum. (Figure 1A). FNAC was performed at the site using a 21-gauge needle (Figure 1B). The cytological findings showed a mixed lymphocytic population alone. An en bloc resection of the lymph node was conducted (Figure 2A,B). The specimen consisted of a grayish and soft mass measuring about 1.0 × 0.7 × 0.6 cm ^3^. The section of the specimen displayed a preserved nodal architecture with reactive follicular hyperplasia and the presence of small clusters of epithelioid histiocytes at the periphery of germinal centers in the affected lymph node (Figure 3). After the pathological report was available, we further completed the relevant serological tests, which revealed elevated levels of *Toxoplasma* IgG (513.70 IU/mL; normal: <1.6 mg/L) and *Toxoplasma* IgM (1.46 index; normal: <0.5 index). TL of the head and neck region was confirmed.

Based on the histopathological report and serological results, an infection specialist prescribed pyrimethamine (25 mg/tab) to treat the *T. gondii* infection. The postoperative surgical wound healed well. The patient’s *Toxoplasma* IgG and IgM levels returned to the normal range at the completion of the treatment course.

## 3. Discussion

TL is a worldwide zoonosis caused by the protozoan parasite *T. gondii* [3,4]. We report a patient with TL of the head and neck region diagnosed using FNAC, serological tests, and histopathological findings.

*Toxoplasma gondii* is estimated to infect about 50% of the population in the United States [5] and Europe [6]. The three transmission routes include food origin (consumption of meat infected with *Toxoplasma* cysts), animal-to-human infection (intake of oocysts shed in the feces of infected cats), and mother-to-fetus infection (congenital infection during pregnancy) [1]. Additionally, *T. gondii* can be transmitted through blood transfusions or organs transplanted from infected donors. A nationwide study in Taiwan found that the consumption of raw shellfish and raising a cat were independent risk factors for infection of *T. gondii* infection [1]. TL is most commonly seen in older children and young adults and occurs more frequently in females than in males [7]. The seasonal prevalence of TL is highest in the winter months. A retrospective study performed at a Pakistani medical institute found that the most common occupations of people with TL were students (48.8%), soldiers (18.6%), housewives (11.6%), teachers (9.3%), and drivers (6.9%) [8].

The majority of *T. gondii* infections are asymptomatic and self-limiting in immunocompetent hosts; however, symptomatic disease can occur in immunocompromised individuals [4,5]. The enlargement of single or multiple superficial lymph nodes is the most common sign of acquired toxoplasmosis in humans [7,9]. The most common site of TL is the neck (69.7%) followed by the axillary (6.9%), submental (4.6%), inguinal (4.6%), and supraclavicular (2.3%) lymph nodes, and other areas (11.6%) [8]. In the neck, the posterior area is most commonly affected [5]. TL is usually painless and non-suppurative. Other symptoms may include fatigue, general weakness without a fever or weight loss, diarrhea, increasing tiredness, and myalgias [10]. *Toxoplasma* infections usually occur in nasopharyngeal lymphoid tissue, which is consistent with cervical lymph node involvement without a sore throat [7]. However, if a fever occurs, infectious mononucleosis should be considered and a blood smear examined for lymphocytosis with atypical lymphocytes. Further, prolonged and persistent enlargement of the lymph node(s) for months or years may warrant clinical suspicion of a malignant lymphoma [7]. Ahmad et al. found that in the final pathological diagnosis as TL, the differential diagnosis clinically included tuberculosis lymphadenitis (55.8%), Hodgkin’s disease (16.2%), malignant lymphoma (11.6%), chronic lymphadenitis (4.6%), infectious mononucleosis (2.3%), reactive changes (2.3%), and no specific diagnosis (6.9%) [8].

Several imaging tools have been shown to be useful for the diagnosis of TL, including US-guided FNA, CT, and magnetic resonance imaging (MRI). US allows the visualization of homogeneous, well-defined enlarged lymph nodes. Kojima et al. found FNAC to be a simple, safe, economical, and quick assessment procedure for TL [11]. Conversely, Cho et al. recommended core needle biopsy (CNB) as the first-line investigation for lymphadenopathy [12].

The infected lymph node was enlarged; however, there was nothing distinctive about the macroscopic appearance [7]. Lymphoid hyperplasia accounted for the enlargement [13]. The follicles were large and irregularly shaped with active germinal centers. Pale-staining histiocytes with an eosinophilic cytoplasm were scattered around the pulp of the lymph node and invaded the follicles. These cells were similar to the epithelioid cells of tuberculosis; however, caseation was absent. Moreover, the cells were less sharp and smaller than the collections of cells seen in sarcoidosis. Histiocytic clusters are characteristic of TL [7]. Weiss et al. noted that a pathological findings in lymph nodes with TL often contain the characteristic triad of reactive follicular hyperplasia, irregular clusters of epithelioid histiocytes (blurring the germinal centers), and focal trabecular and subcapsular sinuses distended by monocytoid B cells [5].

TL can be confirmed by serological tests in the majority of patients and is a primary method for diagnosis [4]. Increasing titers of cytoplasm-modifying antibodies can be considered an active infection [7]. Contini et al. noted that elevated IgM antibody titers were indicative of an acute infection [4]. In fact, the positive serological findings have shown to be sufficient for the diagnosis of TL [7]. PCR has not been used to definitively diagnose TL, although, in some reports, it was applied to biopsy material. 

Pyrimethamine and sulfadiazine remain the first-line treatments for toxoplasmosis.

Trimethoprim–sulphamethoxazole is an alternative medication for patients who cannot tolerate pyrimethamine and sulfadiazine [2].

## 4. Conclusions

We detected TL in the head and neck region of a patient presenting with an enlarged and painless mass. US with FNA should be considered as the first-line test for assessing a mass before a definitive treatment is chosen. Although the diagnosis of TL in the head and neck region is challenging, the combined use of FNAC, biopsy, and serological tests will help clinicians achieve an accurate result.

## Data Availability

All data generated or analyzed during this study are included in this published article.

## Abbreviations

TL = toxoplasmic lymphadenitis; FNAC = fine needle aspiration cytology; US = ultrasound; CT = computed tomography; PCR= polymerase chain reaction.
